# Metabolic engineering of *Streptomyces* to enhance the synthesis of valuable natural products

**DOI:** 10.1016/j.engmic.2022.100022

**Published:** 2022-04-23

**Authors:** Zuwei Xu, Lihao Ji, Wenxiu Tang, Liang Guo, Cong Gao, Xiulai Chen, Jia Liu, Guipeng Hu, Liming Liu

**Affiliations:** a State Key Laboratory of Food Science and Technology, Jiangnan University, Wuxi 214122, China; b School of Life Sciences and Health Engineering, Jiangnan University, Wuxi 214122, China

**Keywords:** *Streptomyces*, Natural products, Metabolic engineering, Life cycle, Biosynthetic gene clusters

## Abstract

The mycelial bacterium *Streptomyces* is a workhorse for producing natural products, serving as a key source of drugs and other valuable chemicals. However, its complicated life cycle, silent biosynthetic gene clusters (BGCs), and poorly characterized metabolic mechanisms limit efficient production of natural products. Therefore, a metabolic engineering strategy, including traditional and emerging tools from different disciplines, was developed to further enhance natural product synthesis by *Streptomyces*. Here, current trends in systems metabolic engineering, including tools and strategies, are reviewed. Particularly, this review focuses on recent developments in the selection of methods for regulating the *Streptomyces* life cycle, strategies for the activation of silent gene clusters, and the exploration of regulatory mechanisms governing antibiotic production. Finally, future challenges and prospects are discussed.

## Graphical abstract

**Figure:**
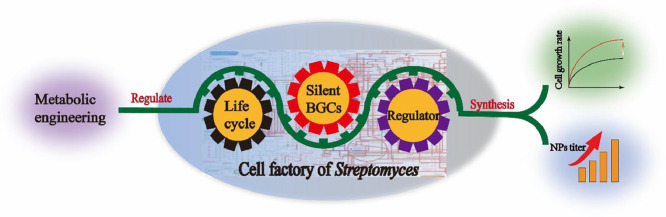

## 1 Introduction

Natural products (NPs) are secondary metabolites that exert specific physiological functions and are produced or secreted by animals, plants, and microorganisms [1]. The molecular structure of NPs varies substantially, which is reflected in the high percentage of NPs and their derivatives (30–66%) being sourced as active drug molecules with anticancer, antifungal, antibiotic, and immunosuppressant properties [2], [3], [4]. In addition, many NPs and their analogs have been developed and widely used as spices, dyes, food additives, pesticides, and veterinary drugs. Given the complexity of animals and plants, their long metabolic life cycles, and the ensuing cost of extracting NPs, microorganisms have become the source of approximately 35% of NPs. Typical microbial producers include bacteria, actinomycetes, and fungi. More than 100 fungal species have been found to produce bioactive substances of pharmacological relevance. *Streptomyces* has emerged as the workhorse for producing valuable NPs, including all major antibiotics [5,6]. Streptomycin is the second most produced and used antibiotic in the clinic and is effective in tuberculosis treatment. Tetracycline is a wide-spectrum antibiotic used extensively to combat infections caused by Gram-positive and Gram-negative bacteria, intracellular mycoplasma, Chlamydia, and *Rickettsia* [7,8]. Nevertheless, there are limitations to the industrial production of antibiotics by *Streptomyces*. First, *Streptomyces* is a mycelial bacterium characterized by a complex developmental life cycle, including vegetative hyphae, aerial hyphae, and spores, which prolongs the fermentation time necessary to synthesize NPs [9]. Second, 90% of biosynthetic gene clusters (BGCs) in *Streptomyces* are silent or cryptic under standard laboratory growth conditions, inhibiting antibiotics output [10]. Third, the regulation of antibiotic metabolic networks in *Streptomyces* remains unclear [11].

With the rapid development of genome sequencing technology, the combined use of omics and systems biology has promoted the analysis of synthetic pathways and mining of key enzymes in *Streptomyces*. Applying bioinformatics, metabolic engineering, and synthetic biology could further increase the efficiency of NP synthesis. In this review, recent advances in *Streptomyces* metabolic engineering, involving optimization of its life cycle, activation and discovery of cryptic BGCs, and exploration of regulatory mechanisms are summarized*.*

## 2 Regulatory strategies for optimizing the *Streptomyces* life cycle

**Fig. 1:**
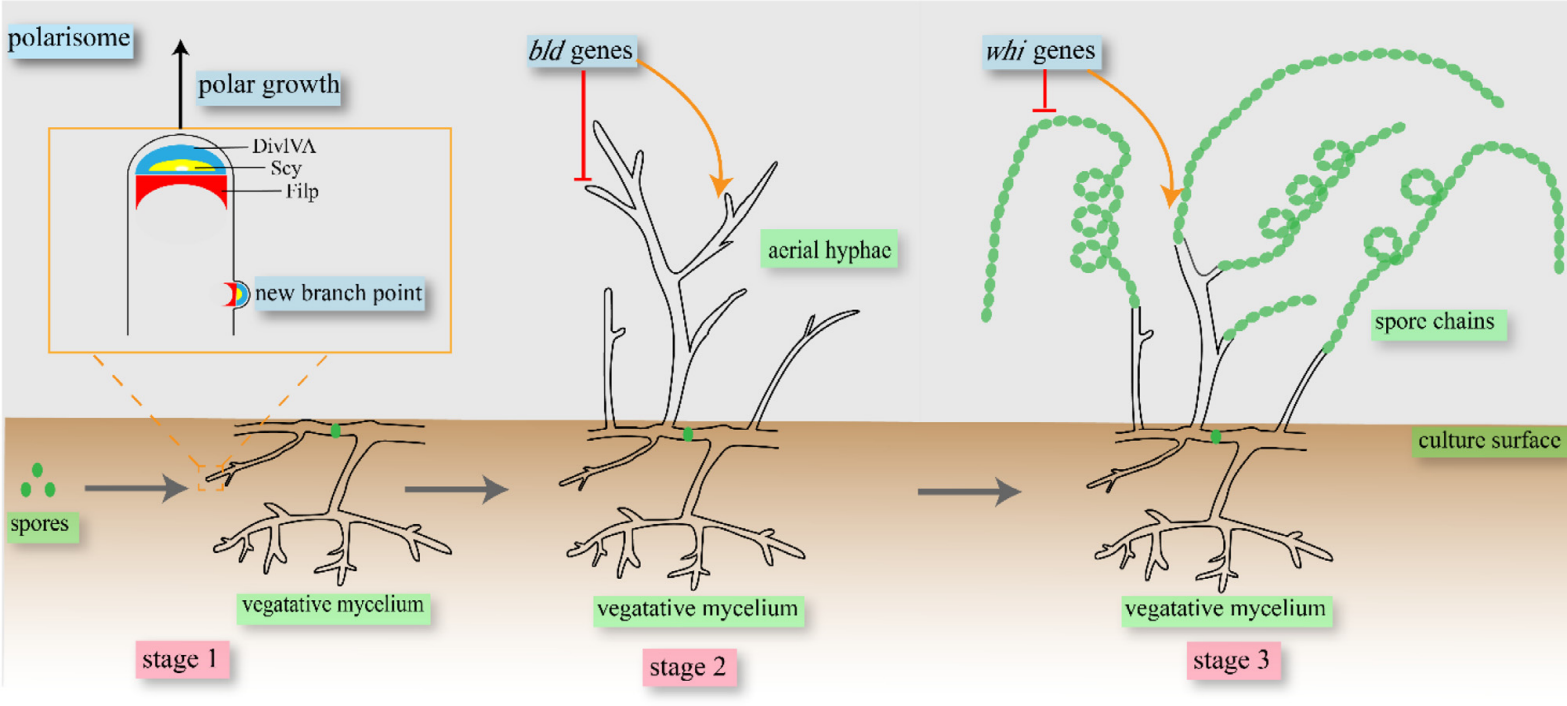
The *Streptomyces* life cycle starts with spores, from which one or two germ tubes emerge. The germ tubes extend by polar growth, new branches appear at sites behind the tip, and the branches develop into a dense network of hyphae, which will form the vegetative mycelium (stage 1). The vegetative mycelium differentiates into aerial hyphae once nutrients are consumed (stage 2). Finally, aerial hyphae differentiate into long chains of spores (stage 3). The three stages can be manipulated by regulating the polarisome, *bld* gene family, and *whi* gene family.

The *Streptomyces* life cycle starts with spores, from which one or two germ tubes emerge. The germ tubes extend by polar growth, while new branches appear at sites behind the tip, where they develop into a dense hyphal network that will form the vegetative mycelium. Once nutrients are consumed, vegetative mycelia differentiate into aerial hyphae, which in turn differentiate into long chains of spores [12]. The life cycle of *Streptomyces* comprises three stages: vegetative hyphae, aerial hyphae, and spores (Fig. 1). To shorten the fermentation time required for NP synthesis via growth cycle modulation, research has focused on key regulatory genes with wide-ranging effects on *Streptomyces* development and nucleotide second messengers participating in its life cycle transitions [13].

### 2.1 Key regulatory genes responsible for *Streptomyces* morphogenesis

The three stages in the *Streptomyces* life cycle can be manipulated through the polarisome complex or the *bld* and *whi* gene families. The vegetative hyphae stage dominates polar growth under control of the polarisome complex, which is comprised of three coiled-coil proteins: DivIVA, Scy, and FilP (Table 1). DivIVA is the main constituent of the polarisome and is required for branch polar elongation, cell shape acquisition, and directing the polar cell wall in *Streptomyces*. Branch polar elongation may be the most important function of DivIVA because it can determine growth direction, curvature, and cell width. *divIVA* overexpression in *Streptomyces coelicolor* A3(2) can strongly perturb cell shape and cause hyper-branching, with branch tips becoming swollen, rounded, and appearing to grow out along the length of hyphae [14].

**Table 1:** Genes involved in morphologic development.

Gene	Protein	Known function	References
BSU28030	MreB	Coordinate cell wall assembly	[82]
spr1505	DivIVA	Direct polar cell wall synthesis	[83]
SCY-1027	Scy	Stabilize the polarisome	[15]
SSP35-01-08470	FilP	Affect peptidoglycan assembly and cell shape	[84]
SCO1675	BldA	A global regulator	[85]
SSP35_05_03540	BldN	Effect the formation of the mycelia	[86]
SSP35_05_00750	BldM	Regulate the formation of aerial hyphae	[87]
HCU77_07280	BldD	A master developmental repressor	[88]
GTZ89_43905	BldC	Maintain the vegetative growth	[22]
SSP35_04_00670	WhiI	Regulate the differentiation of reproductive structures into mature spores	[87]
SCO3034	WhiB	Control the formation of the spores	[89]
SCO1950	WhiA	Effect the differentiation of aerial hyphae into spores	[90]

Scy is a scaffold protein that stabilizes the polarisome and interacts with DivIVA to maintain normal polar growth. The best known function of Scy is controlling apical dominance [15]. In the *scy* deletion mutant, about 33% of new branch points were in the tip-proximal region, which caused the resulting colonies to be one-half the diameter of their wild-type counterparts [15]. FilP is a cytoskeletal protein that is not necessary for cell growth, but its mutation affects hyphal morphology because FilP provides rigidity and elasticity to the hyphal tip. Accordingly, hyphae of the *filP* deletion mutant become more curved and curled, and do not maintain a normal growth direction [16]. Spore germination represents a system of transformation from the metabolic zero point to a new life cycle. The regulation of spore germination is significant for optimizing the *Streptomyces* life cycle. In many cases, resuscitation promoting factor (Rpf) enzymes are required to resuscitate metabolically quiescent spores. Generally, losses of *rpf* genes were correlated with delayed onset of germination [17]. To promote spore germination, Danielle and co-workers encoded three PASTA domain-containing protein kinases in *S. coelicolor* and obtained a triple mutant strain. The results showed that germination and growth of the triple mutant strain was consistently more rapid than the parent strain [18].

The *bld* gene family controls the aerial hyphae stage, which is primarily responsible for regulating aerial mycelia formation (Table 1) [19,20]. Interventions in *bld* genes block aerial hyphae from entering development and induce precocious sporulation in the vegetative mycelium. Within the *bld* gene family, BldD is a major developmental repressor that prevents sporulation during aerial growth. BldD represses almost 170 sporulation genes during vegetative growth, leading to the growth of “bald” colonies, which lack the fuzzy wild-type appearance. BldD also functions as an on-off switch regulating the transition from morphological differentiation to secondary metabolism. In *Streptomyces lincolnensis*, deletion of *bldD* blocked sporulation, which reduced production of lincomycin, while the morphological phenotype and lincomycin production were restored when a functional *bldD* gene was introduced into the *bldD* deletion mutant. This result showed that BldD directly regulates spore formation and controls lincomycin biosynthesis in *S. lincolnensis* [21]. In contrast, BldC is an activator responsible for promoting the recovery of sporulation in vegetative mycelium. Bush et al. constructed a *bldC* deletion mutant strain of *Streptomyces venezuelae* to determine the relationship between BldC transcription factors and *Streptomyces* differentiation. Whereas the wild-type and *bldC* mutant looked similar (vegetative growth only) after one day, the former produced aerial hyphae after two days, while the latter was still restricted to vegetative growth. Intriguingly, after three days, both strains produced spores, suggesting that the *bldC* mutant entirely bypassed aerial mycelia formation, and almost the entire biomass differentiated into spores [22].

The spore stage is encoded by the *whi* gene family (Table 1), which directly controls the cessation of aerial hyphae division and guarantees sporogenic cell division at the appropriate time. In the early stages of sporulation, mutations in *whiA* prevent *Streptomyces* from synthesizing the green polyketide pigment associated with fully developed spores, resulting in formation of unusually long aerial hyphae that fail to initiate septation or chromosome segregation. Presently, it is unclear how *whi* genes control spore formation. In recent years, *whi* has been proposed to guarantee timely transcription of the spore-division gene *ftsZ*. In *S. coelicolor*, deletion of *whiA* resulted in excessive accumulation of FtsZ, which led to 10% fewer spores compared to the wild type. These results indicate that *whi* ensures sporulation-specific cell division only once sufficient aerial mycelium biomass has been generated [23].

### 2.2 Nucleotide second messengers regulate transitions in the *Streptomyces* life cycle

The nucleotide second messengers cyclic diguanylate (C-di-GMP), cyclic diadenylate (C-di-AMP), and guanosine pentaphosphate and guanosine tetraphosphate (collectively referred to as (p)ppGpp) are crucial regulators of the *Streptomyces* life cycle. c-di-GMP is involved in regulating a wide range of cellular functions and is crucial for signaling initiation and termination of development. Specifically, it controls RsiG, which sequesters σ^WhiG^ and prevents aerial hyphae from forming long chains of spores. Thus, deleting *rsiG* causes vegetative hyphae to differentiate into spore chains, meaning that *Streptomyces* biomass is comprised almost entirely of spores [24]. c-di-AMP controls cell wall synthesis, metabolism, virulence, DNA integrity [25], and cellular integrity. Latoscha et al. constructed a c-di-AMP hydrolase deletion mutant strain, *ΔataC*, which exhibited a severe delay in development. The *ΔataC* strain developed aerial hyphae only after four days of culture, while the wild-type displayed fully sporulated hyphae [26]. These results suggest that elevated levels of c-di-AMP impair cell growth and development.

During exponential growth, (p)ppGpp is a major regulator of cell growth and metabolism, because it inhibits production of ribosomal RNA and transcription of growth-related genes under nutrient-limiting and stressful conditions. [27] A total of 598 genes, including those for ribosome production and function, carbon metabolism, oxidative phosphorylation, cell wall, and fatty acid biosynthesis, were significantly affected in a ppGpp null strain, causing delayed formation of sparse aerial mycelia and no spores or pigment [28]. In another study, by analyzing mutants with impaired abilities to elicit a stringent response, ppGpp was shown to function in triggering antibiotic production in *Streptomyces*. Hesketh et al. compared ppGpp+ and ppGpp- strains to observe the differences in gene expression, and discovered that ppGpp+ activates gene transcription associated with secondary metabolic processes. This result indicates that ppGpp synthesis is required to trigger the onset of antibiotic production in *S. coelicolor* [29]*.* The *Streptomyces* life cycle has been deeply investigated, but there are still some unclear mechanisms of the transition from morphological differentiation to the production of secondary metabolism. Once these mechanisms have been elucidated, the efficiency of producing NPs can be enhanced by regulating these transition switches.

## 3 Strategies for the activation of silent gene clusters

**Fig. 2:**
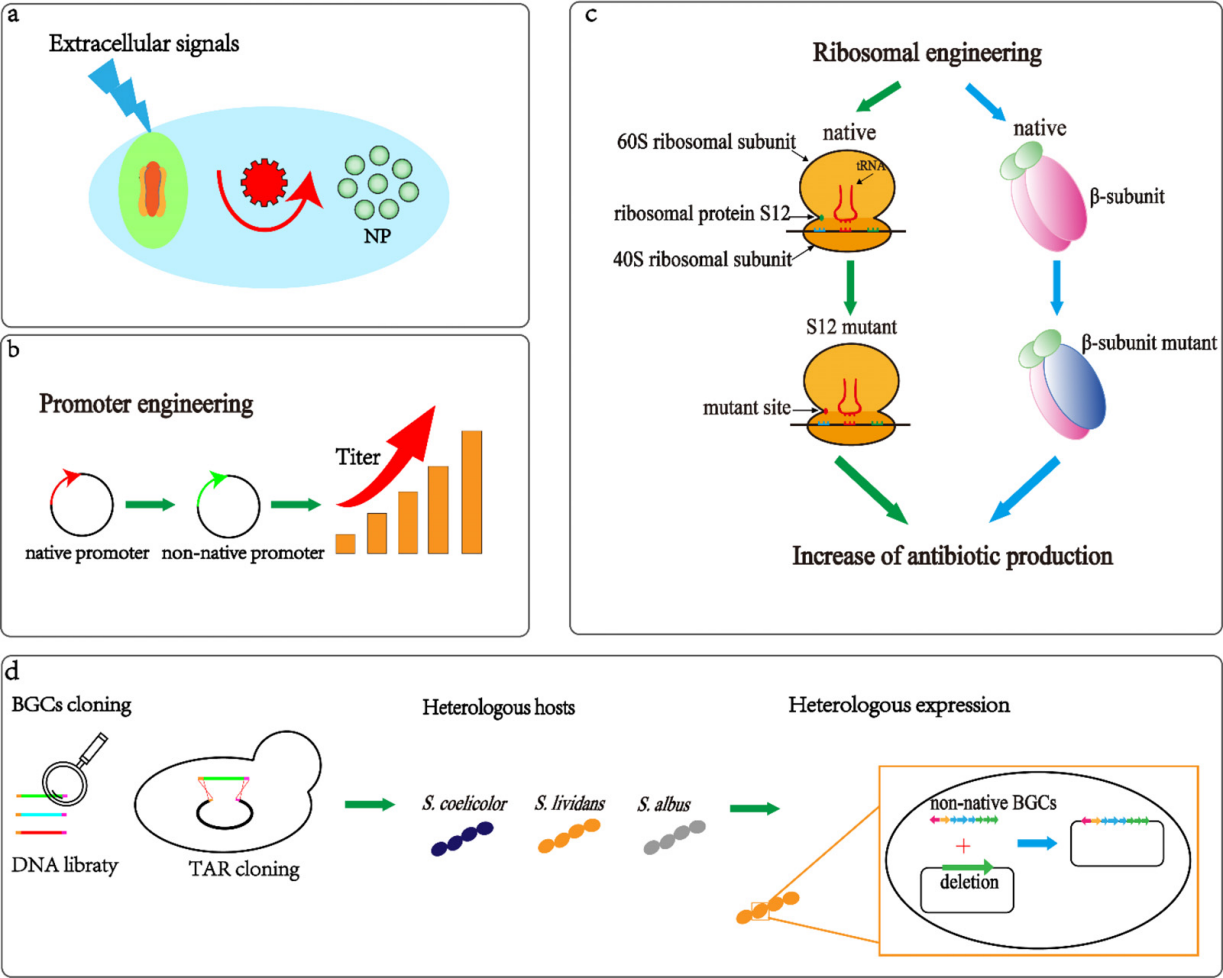
Strategies for the activation of silent gene clusters: (a) use of extracellular signals; (b) promoter engineering; (c) ribosomal engineering; and (d) heterologous expression of silent BGCs.

*Streptomyces* exhibits strong biosynthetic capacity, which has led to 70% of NPs being obtained from this genus. The *Streptomyces* genome contains 25–50 BGCs [30]; however, almost 90% of them are silent or cryptic under standard laboratory growth conditions. This presents a challenge for discovery of novel NPs or for increased synthesis of existing ones. Therefore, activation and regulation of silent and cryptic BGCs have become the focus of recent efforts in NP production [31]. Existing methods for activating silent gene clusters include (1) extracellular signals, (2) reconstruction of NP biosynthetic pathways, and (3) heterologous expression of silent BGCs (Fig. 2).

### 3.1 Extracellular signals activate silent BGCs

The yield of NPs is affected by environmental conditions and nutrient availability. An efficient strategy for enhancing NP output or identifying novel NPs is to use extracellular signals, such as environmental stressors and hormone-like signaling molecules. Environmental stressors, including heat shock, pH shock, and high NaCl conditions, have been used to enhance secondary metabolite production by *Streptomyces*. Heat shock is widely used to activate silent BGCs because it triggers a universal cellular response that promotes cell survival at higher temperatures. In one study, the yield of actinomycin C was 27% higher in *Streptomyces* spore suspensions treated at 50°C for 10 min than in untreated samples [32]. Using pH shock to activate silent BGCs relies on enhancing proton transport and cell respiration, which provides more ATP for cell growth. Alkaline pH shock has been shown to increase validamycin A production by 27.43% in *Streptomyces hygroscopicus* 5008 [33].

Hormone-like signaling molecules are diffusible small compounds that prompt global regulators to activate transcription of numerous genes required for secondary metabolism and morphological differentiation. Self-regulated signaling via one of the more than 14 γ-butyrolactones has been reported in at least 60% of *Streptomyces* species. Tan et al. added the γ-butyrolactone analog 1,4-butyrolactone to culture broth of validamycin A-producing *Streptomyces*, resulting in a 30% increase in the antibiotic titer in both the shake flasks and bioreactors [34]. Notably, avenolide is necessary for triggering avermectin production. Four different avenolide-like compounds were identified in *Streptomyces albus*, and were applied in *Streptomyces avermitilis* to strongly enhance production of the novel avermectin derivatives B2a, A2b, A2a, B1a, A1b, and A1a [35]. To date, hormone-like signaling molecules have been widely used in screening high-yield production strains of natural products. Still, these classical activation methods are limited by various factors, including being time-consuming and labor intensive. Therefore, an efficient high-throughput screening strategy is urgently required. However, the construction of such a system for *Streptomyces* is challenging, due to complex cellular structures and because quantifying the target compounds is difficult.

### 3.2 Reconstruction of the BGC pathway in native hosts

Another strategy for activating silent BGCs is reconstructing their biosynthetic pathways; however, this is hindered by the complex regulatory mechanism controlling BGCs. To overcome this problem, promoter and ribosomal engineering have been applied. Promoter elements are responsible for efficient transcription, which is of great interest for activating silent genes [36]. Promoters can be either constitutive or inducible. A strong constitutive promoter is *Psco5768*, which was identified in the genome sequence of *S. coelicolor* M145. When placed upstream of *jadJ* to drive the jadomycin BGC, the jadomycin B titer increased two-fold [37]. Replacing the constitutive promoters in actinorhodin-producing *S. coelicolor* M145 with a cumate inducible promoter increased the titer of actinorhodin by three-fold under the optimum induction time and dosage (35 h and 2.5 μM cumate) [38]. This result shows that manipulating the trade-off between product biosynthesis and other physiological events using an inducible promoter can effectively improve the titer in *Streptomyces*.

Ribosomal engineering, including traditional mutagenesis [39] and rational metabolic engineering, is another efficient approach for activating silent BGCs. In traditional mutagenesis, a mutant resistant to antibiotics and capable of simultaneously increasing the amount of target product is selected [40]. A typical example is establishing a novel method to activate ε-poly-L-lysine synthesis, which combined atmospheric and plasma mutagenesis with six drug-resistance mutations. The resulting R6 mutant resistant to six antibiotics produced 4.41 g L^−1^ of ε-poly-L-lysine in a shake flask, which was 2.75-fold more than the parent strain [41]. In metabolic engineering, the versatile ‘transcription and translation in one’ strategy was designed to activate the silent BGCs associated with a cyclized derivative of undecylprodigiosin and identified from the genome of *Streptomyces lividans* TK21. Overexpression of exogenous *rpsL* (encoding ribosomal protein S12) and *rpoB* (encoding RNA polymerase β subunit) genes in *S. lividans* TK2 led to successful identification of the new compound streptorubin B [42].

### 3.3 Heterologous expression of silent BGCs

Reconstructing the BGC biosynthetic pathways in native hosts is an efficient approach to unlock silent BGCs; however, there are some limitations to its applications. First, the expression of BGCs in the native host strain may lead to slow or unmanageable growth under fermentation conditions. Second, only a limited set of genetic manipulation tools exist in *Streptomyces*. Third, scant physiological knowledge of *Streptomyces* impedes heterologous reconstruction of BGCs.

Widely used heterologous host strains include *S. coelicolor, S. lividans*, and *S. albus*. These strains satisfy the following requirements: (1) they can be easily transformed and genetically manipulated; (2) they allow efficient expression of heterologous BGCs; and (3) they contain sufficient precursors for targeted NPs [43]. *S. coelicolor* yields a wide range of NPs owing to a diverse range of synthetic biology tools [44,45], which makes it the preferred choice of the three. As *S. lividans* can accept methylated DNA, it is a suitable host strain for the heterologous production of various recombinant proteins [46]. *S. albus* has been widely used as a heterologous host strain for the expression of silent and cryptic BGCs to produce potent anticancer agents [47]. Targets include steffimycin biosynthetic genes [48], as well as fredericamycin, isomigrastatin, napyradiomycin, cyclooctatin, and thiocoraline BGCs [49], [50], [51].

Heterologous reconstruction of targeted BGCs in the host strain can be achieved via either traditional library construction or transformation-associated recombination (TAR). Traditional library construction is used to clone small BGCs (<40 kb). A BGC associated with actinoallolide was found by constructing a cosmid library from the *Actinoallomurus fulvus* K09-0307 genome and then successfully expressing it in *S. coelicolor* M1152. Subsequent liquid chromatography-mass spectrometry revealed novel products in the culture broth, including actinoallolide A and ethylacetate [52]. Given that traditional techniques are often time consuming and laborious, TAR was introduced to further increase construct efficiency. TAR is a powerful and reliable method to directly clone large BGCs. Through this method, which involves regulatory gene remodeling, it was possible to successfully express a 67-kb non-ribosomal peptide synthetase BGC from the marine actinomycete *Saccharomonospora* sp. CNQ-490 and produce the dichlorinated lipopeptide antibiotic taromycin A in *S. coelicolor* [53].

Heterologous expression of silent or cryptic BGCs has two aims: increase NP yields and identify novel compounds. Daptomycin is a Food and Drug Administration (FDA)-approved, highly valuable antibiotic that exhibits strong bioactivity against gram-positive pathogens. The 65-kb daptomycin BGC from the *Streptomyces roseosporus* genome was cloned directly in *S. coelicolor* M511 using a BAC library, leading to a ten-fold higher daptomycin titer compared to the parent strain [54]. In another example, an orphan silent *lav* gene cluster from the genome of *Streptomyces lavendulae* FRI-5 was identified by Illumina sequencing, and a new diol-containing polyketide called “lavendiol” was found when this gene cluster was heterologously expressed in the engineered strain *S. avermitilis* SUKA22 [55]. Currently, these strategies combine functional genomics and bioinformatics to identify the products of activated BGCs, such as novel pyranonaphtoquinones [56], deoxacalcimycin [57], and surugamide A [58]. Unfortunately, due to the high investment and low return rate of activating silent BGCs, the discovery of new natural products has entered a bottleneck. In the future, artificial intelligence can be applied in genetic studies and for metabolic profiling. Once a further development metabolic engineering strategy is obtained, activation of silent BGCs will develop more rapidly.

## 4 Regulatory mechanisms of antibiotic production

**Fig. 3:**
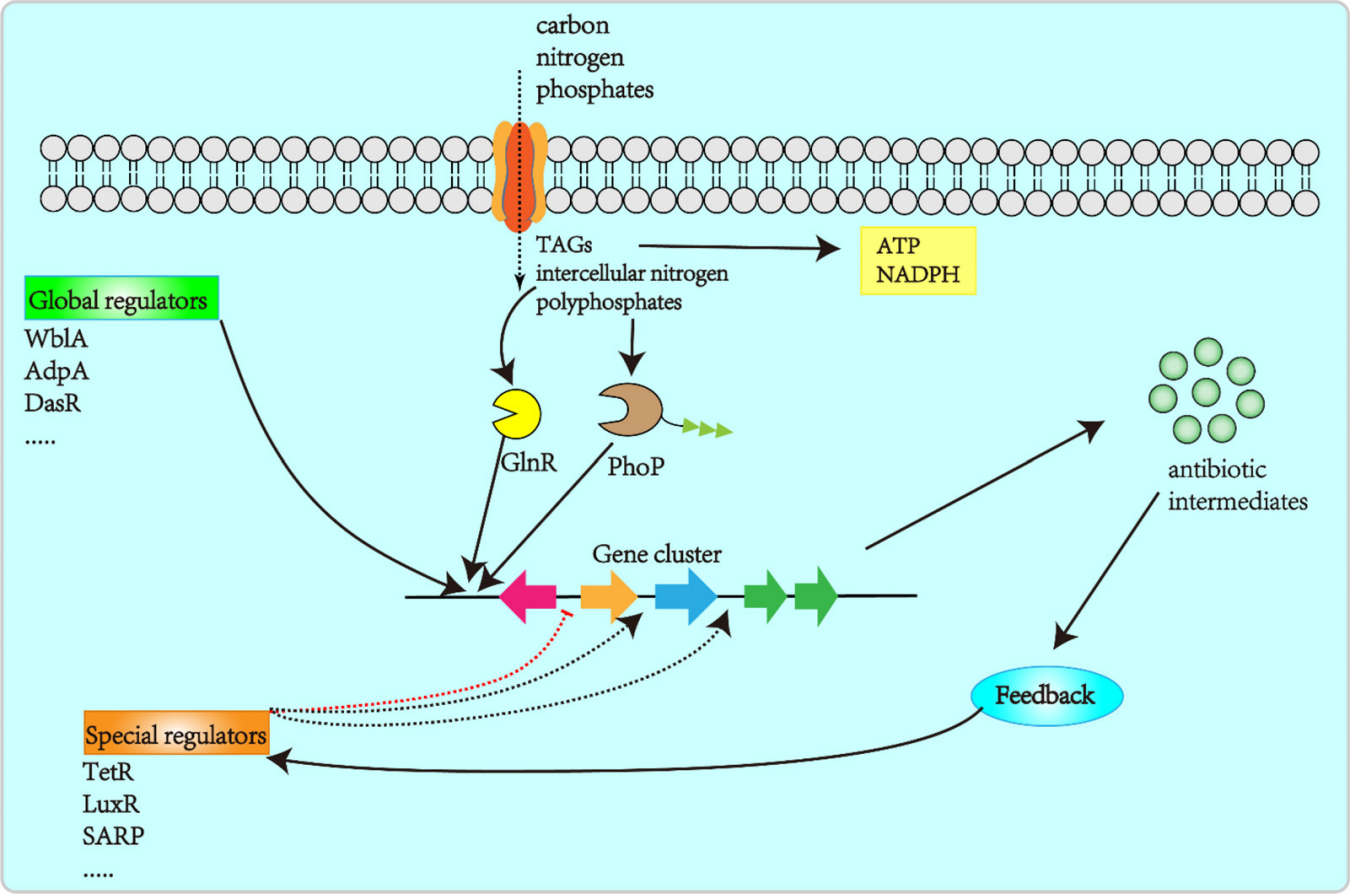
Schematic representation of the regulatory cascades controlling antibiotic production in *Streptomyces.*

The production of metabolites in *Streptomyces* is tightly controlled by pyramidal transcriptional regulatory cascades [59]. The synthesis of NPs occurs in two stages: (1) during primary metabolism, as cells consume external nutrients and grow rapidly; and (2) when external nutrients become limited, cell growth ceases, and secondary metabolites start being produced [60]. The mechanism guiding the passage from cell growth to secondary metabolite production remains poorly understood. In recent years, scientists have worked to identify metabolic switches from primary to secondary metabolites. In this section, we summarize recent advances in this metabolic switching, including key intracellular metabolites, global and pathway-specific regulators, and the feedback regulatory switch (Fig. 3).

### 4.1 Dynamic regulation of key intracellular metabolites

Three key intracellular metabolites—triacylglycerols, nitrogen, and polyphosphates—were found to dynamically manipulate the metabolic flux towards targeted secondary metabolites in *Streptomyces* during the stationary phase. In *Streptomyces*, triacylglycerols are the main components of cellular lipids and act as an intracellular carbon source for antibiotic production [61]. Degradation of the cellular triacylglycerol pool can produce more acetyl-CoA building blocks and generate a higher NADH/NAD^+^ ratio and more ATP. Thus, rational regulation of triacylglycerol metabolism could increase the intracellular level of acetyl-CoA, while lowering the NADH/NAD^+^ ratio and ATP content during the stationary phase, thus directing the carbon flux towards polyketide synthesis. A ‘dynamic degradation of triacylglycerol’ strategy was designed to fine-tune triacylglycerol mobilization, substantially increasing the yields of actinorhodin, jadomycin B, oxytetracycline, and avermectin B1a in *S. coelicolor, S. venezuelae, Streptomyces rimosus*, and *S. avermitilis* [62].

Glutamate and glutamine are the major amino acid donors in transamination reactions that generate nitrogen-containing compounds [63]. GlnR is a central regulator of nitrogen metabolism in *Streptomyces*, which directly guides the transition from primary metabolism to antibiotic biosynthesis [64]. *S. avermitilis* can produce avermectin and oligomycin; interestingly, a *glnR* null mutant drastically reduced the avermectin titer but promoted the biosynthesis of oligomycin [65]. Given that polyphosphates are essential substrates for NP biosynthesis, some NPs are synthesized upon phosphate limitation [66]. PhoP is a major regulator of phosphate metabolic genes in *Streptomyces*. Under phosphate-limited conditions, PhoP down-regulates genes involved in aerobic respiration, up-regulates genes involved in nitrate respiration, delays cell development, and triggers NP synthesis [67,68]. Deletion of the *ppk*/*pstS* genes, encoding polyphosphate kinase and the high-affinity phosphate-binding protein PstS, was found to reduce intracellular storage of polyphosphate in *S. lividans*, while promoting inorganic phosphate intake from phosphate-limited medium. The same double mutant expressed up to ten-fold more endogenous actinorhodin and heterologous 8-demethyl-tetracenomycin C than the wild-type strain under phosphate limitation [69,70].

### 4.2 Global and pathway-specific regulators governing antibiotic production

*Streptomyces* harbors various regulators that control NP synthesis, which can be broadly classified as pathway-specific or global. Pathway-specific regulators are usually located within specific BGCs and affect the expression of gene clusters. They comprise TetR, SARP, LuxR, LysR, and MarR family regulators, and are based on their amino acid sequences and functions (Table 2) [71]. Regulators of the TetR family in some cases play a negative role in NP biosynthesis. In *S. avermitilis*, the TetR family regulator AccR represses production of malonyl-CoA and methylmalonyl-CoA, which are precursors of avermectin B1a. Deletion of *accR* led to a higher titer of avermectin B1a (304 μg mL^−1^) compared to the wild-type (190 μg mL^−1^). This result shows that AccR indirectly controls avermectin B1a production by regulating the acyl-CoA pool [72]. In contrast, the SARP family regulator OtcR activates five *oxy* promoters to enhance oxytetracycline biosynthesis, as indicated by an increase of the oxytetracycline titer to 6.24 g L^−1^ following optimized expression of *otcR* in *S. rimosus* M4018::SFotcR [73].

**Table 2:** Well-studied regulators involved in antibiotic production.

Regulator type	Strain	Known function	References
Pathway-specific regulators			
TetR	*S. avermitilis*	Activator for avermectin	[72]
LuxR	*S. rimosus*	Activator for oxytetracycline	[72]
SARP	*S. aureofaciens*	Activator for chlortetracycline	[91]
LysR	*S. globisporus*	Effect the formation of the mycelia	[86]
MarR	*S. coelicolor*	Activator for chlortetracycline	[92]
Global regulators			
WblA	*S. coelicolor*	Antibiotic downregulator	[75]
AdpA	*S. coelicolor*	Central transcriptional regulator	[76]
DasR	*S. griseurs*	Regulator of secondary metabolite gene expression in response	[93]
AtrA	*S. globisporus*	Transcriptional activator of actinorhodin;	[94]
RelA	*S. coelicolor*	ppGpp synthetase gene	[95]

Unlike pathway-specific regulators, global regulators are located outside BGCs and do not directly control the expression of specific BGCs, instead affecting NP synthesis by regulating morphological differentiation and the primary metabolic flux in *Streptomyces* (Table 2). They include WblA, AdpA, DasR, AtrA, and RelA [74]*.* WblA, a WhiB-like protein first identified in *S. coelicolor*, is a global repressor that controls antibiotic biosynthesis and morphological differentiation. WblA was found to lower the production of daptomycin by down-regulating genes with a positive regulatory effect on daptomycin and silencing daptomycin biosynthetic genes in *Streptomyces peucetius*. Deletion of *wblA* resulted in a 51% higher daptomycin titer compared to the wild-type strain, together with loss of the ability to form spores or long-chain spore structures [75].

AdpA, a regulator of the AraC/XylS family, is ubiquitous in *Streptomyces*. AdpA is a transcriptional activator of antibiotic production. The *Streptomyces chattanoogensis* ortholog of AdpA, AdpAch, positively regulates natamycin biosynthesis by affecting two natamycin production pathway-specific genes, *scnRI* and *scnRII*. First, AdpAch binds to the *scnRI–scnRII* intergenic region to trigger the transcription of *scnRI* and *scnRII*. Then, feedback via ScnRI activates *scnRI* and *scnRII* transcription. Based on this series of events, Yu and co-workers randomly mutated six AdpAch binding sites in the *scnRI–scnRII* intergenic region (Sites A to F), and found that the natamycin yield was 21% and 25% higher in the R-mA (mutation in Site A) and R-mF (mutation in Site F) strains, respectively, compared to that in wild-type [76].

### 4.3 Feedback regulation of intermediates or antibiotics

In *Streptomyces*, metabolic precursors and intermediates of NPs affect NP synthesis through feedback regulation of enzymatic reactions and expression of the corresponding biosynthesis genes [77]. A self-regulating and fine-tuned strategy was developed to balance distribution of the ketene precursor between polyketide biosynthesis and other physiological needs. The resulting titers of actinorhodin and oxytetracycline were respectively 1.3-fold and 1.9-fold higher in the engineered strains *S. coelicolor* M145 and M1146^38^, compared to the wild-type. This finding shows that precursors can serve as feedback modulators for enzymatic reactions. However, precursors and intermediates can also serve as self-regulatory molecules for concerted production of antibiotics [78]. The calcimycin biosynthetic intermediate cezomycin can act as a repressor ligand to induce CalR3 dissociation from its binding sites during calcimycin biosynthesis in *Streptomyces chartreusis* NRRL 3882. Moreover, CalR3 can entirely lose its DNA-binding affinity when the cezomycin content is increased to 1.9 mM [79]. These examples suggest that timely coordination of NP biosynthesis with a supply of precursors and intermediates can benefit product synthesis.

With increasing NP content in the fermentation broth, the production rate tends to decrease due to product inhibition. To overcome this issue, NP resistance genes can be targeted for genetic manipulation. The oxytetracycline BGC contains three genes, *otrA* (ribosomal protection protein), *otrB* (efflux proteins), and *otrC* (efflux proteins), which are involved in tetracycline resistance. Among them, OtrA is a ribosomal protection protein that may prevent binding of oxytetracycline to its inhibitory sites on ribosomes. OtrB and OtrC are efflux proteins that export intolerable oxytetracycline to protect the host strain from attack by continually synthetized oxytetracycline. Simultaneous overexpression of *otrA, otrB*, and *otrC* improved the resistance to oxytetracycline and increased the oxytetracycline titer to 7.49 g L^−1^ by *S. rimosus* MKRABC [80]. Antibiotics can also serve as self-regulators to control their own biosynthesis pathways, as exemplified by a feedback model for rifamycin export. In the early growth phase, RifQ represses rifamycin B export by accelerating intracellular rifamycin B accumulation. When the intracellular rifamycin B content reaches a threshold, rifamycin B inactivates RifQ to export more rifamycin B and thus protects the host strain from endogenous rifamycin B toxicity [81].

In this section, we summarized recent advances in the regulatory mechanisms of antibiotic production, but the regulatory mechanisms in most cases are still not clear. Therefore, further studies should focus on regulatory knowledge of antibiotic biosynthesis to increase the yields of antibiotics.

## 5 Conclusions

*Streptomyces* has become an essential platform to produce a wide range of valuable secondary metabolites, including antibiotics, anticancer agents, and immunosuppressants. Using current biotechnology strategies, the life cycle of *Streptomyces* can be shortened, the production of NPs can be increased, and the problem of silent gene clusters can be partly solved. These improvements often result in higher NP production, which ameliorates antibiotic resource shortages. Overall, in the past decade, significant process has been made in rational metabolic engineering modification to breed NP production strains, but it still requires a time-consuming and error-prone process. Hence, emerging biological tools such as data science and artificial intelligence need to be increasingly used in upgrading systems metabolic engineering to improve the production of NPs. Once these emerging tools can be better applied in the biosynthesis of NPs, we believe they will contribute to discovering new antibiotics and improving the yields of known antibiotics.

## Declaration of Competing Interest

The authors declare that they have no known competing financial interests or personal relationships that could have appeared to influence the work reported in this paper.
